# Neuronal Intranuclear Inclusion Disease Presenting with Acute-Onset Dementia and Cortical Edema: A Case Report

**DOI:** 10.3389/fneur.2024.1464991

**Published:** 2024-10-28

**Authors:** Xiao Feng, Yue Li, Qin Zhao, Shabei Xu

**Affiliations:** Department of Neurology, Tongji Hospital, Tongji Medical College, Huazhong University of Science and Technology, Wuhan, China

**Keywords:** neuronal intranuclear inclusion disease, dementia, magnetic resonance imaging, NOTCH2NLC gene, neurodegenerative disease, case report

## Abstract

**Background:**

Neuronal Intranuclear Inclusion Disease (NIID) is a neurodegenerative disorder characterized by the formation of eosinophilic inclusions in the neurons, visceral and skin cells. The cause is associated with the GGC nucleotide repeat expansion in the NOTCH2NLC gene. The imaging hallmark of NIID is hyperintensities on diffusion-weighted imaging (DWI) at the corticomedullary junction. Clinical manifestations of NIID are highly heterogeneous. Here, we report a case of NIID presenting with acute-onset dementia and cortical edema.

**Case presentation:**

We describe an elderly male patient who presented with sudden dementia within a day. Considering the abrupt onset and the stroke history, we initially diagnosed vascular disease. However, further imaging revealed cortical edema in the temporo-parieto-occipital lobes. Blood and cerebrospinal fluid tests ruled out immunological, metabolic, infectious, or neoplastic etiologies. Genetic testing ultimately confirmed the diagnosis of NIID. Intravenous immunoglobulin (IVIG) therapy did not improve the patient’s symptoms; However, about 1 month after treatment, spontaneous improvement was observed. It is noteworthy that 22 months before the onset of cognitive impairment, the patient’s MRI for headaches already exhibited the typical imaging lesions of this disease in the cerebellum paravermal region.

**Conclusion:**

Patients with encephalopathy syndrome exhibiting imaging features resembling mitochondrial encephalomyopathy, lactic acidosis, and stroke-like episodes (MELAS) syndrome or Creutzfeldt-Jakob disease should consider the NIID as differential diagnosis. Chronic headaches and symmetric lesions in the cerebellar paravermal region on MRI may be noteworthy indicators of NIID during non-episodic phases.

## Introduction

Neuronal intranuclear inclusion disease (NIID) is a chronic neurodegenerative disease characterized by the formation of eosinophilic inclusions within the nuclei of neurons in both the central and peripheral nervous systems, which can also be present in visceral, muscular, and cutaneous cells (1). As a monogenic mutation disease, NIID is currently speculated to be associated with the amplification of the GGC trinucleotide sequence in the 5′ untranslated region (UTR) of the NOTCH2NLC gene (2). NIID is classified into sporadic and autosomal dominant inheritance types (1). According to the symptom onset, NIID is also categorized into pediatric, adolescent, and adult types, with the adult-onset type being predominant in East Asia (3). Due to the widespread involvement of the nervous system, clinical manifestations of NIID exhibit marked heterogeneity. In children and adolescents patients, the initial presentation usually involves ataxia or psychiatric and behavioral abnormalities, while in adult-onset NIID, the most common initial symptoms are limb weakness or dementia (4). The propensity for the dementia onset is elevated with increasing age of patients (1). Common manifestations also include movement disorders, autonomic neuropathy, peripheral neuropathy (1, 5). Additionally, visual impairment and headaches may be the chronic symptoms of NIID. The definitive diagnosis of NIID can be established through the skin biopsy (1), revealing the presence of inclusions, or by quantifying the GGC repeat expansion in the NOTCH2NLC gene (2).

The characteristic imaging feature of NIID is the hyperintensities in the corticomedullary junction on diffusion-weighted imaging (DWI), also known as the subcortical lace sign (5). Additionally, bilateral symmetric high signals in the white matter, middle cerebellar peduncles and paravermal region on MRI T2-weighted imaging (T2WI) and fluid-attenuated inversion recovery (FLAIR) sequences, also support the diagnosis of NIID (5, 6). We reviewed a NIID patient confirmed by genetic testing, presenting with acute-onset dementia and focal unilateral cortical edema on imaging. The clinical manifestations of this case overlapped with autoimmune encephalitis, metabolic encephalopathy, and central nervous system infections, etc. Notably, the imaging findings lacked the typical subcortical lace sign but resembled conditions such as mitochondrial encephalomyopathy, lactic acidosis, and stroke-like episodes (MELAS) syndrome or Creutzfeldt-Jakob disease (CJD). It is noteworthy that 22 months before the onset, the MRI of the patients already showed the clues of NIID. The rare imaging presentation and diagnostic challenges in this case, along with the lessons from the initial consultation, can provide valuable insights for neurologists in clinical practice.

## Case presentation

A 60-year-old male with a history of ischemic stroke experienced a sudden onset of cognitive decline in a day, manifested by an inability to recall his granddaughter’s name, failure to recognize relatives, and a complaint of headache, numbness in the right limbs and face, and unsteady gait. Upon admission to a local hospital, the CT and MRI revealed multiple lacunar infarcts of his brain. The patient was initially diagnosed with acute cerebrovascular disease and treated with antiplatelet agents and statins. One week later, the cognition decline worsened, marked by a complete inability to recognize relatives and objects, significant deficits in temporal and spatial orientation, and memory decline. Subsequently, he was transferred to our hospital. Upon admission, the patient was uncooperative with the following pertinent physical examination, had clear consciousness, and exhibited reduced limb tendon reflexes and 3 points on MMSE (retaining the ability of orientation to the province, reading, and paper folding tasks).

Sudden-onset cognitive impairment is commonly associated with acute damage to the cerebral cortex or white matter, which can be attributed to vascular, immune, infectious, endocrine, metabolic, neoplastic and toxic causes, etc. The patient had no fever, without history of toxin exposure, hypoxia, radiation therapy, hypoglycemia or alcohol abuse. Blood tests at admission was normal (the complete blood count, D-dimer, liver and kidney function, thyroid function, antithyroid peroxidase antibodies, thyroglobulin antibodies, electrolytes, antibodies for systemic rheumatic immune diseases, heavy metals, folic acid, vitamin B12, and tumor markers).

**Figure 1:**
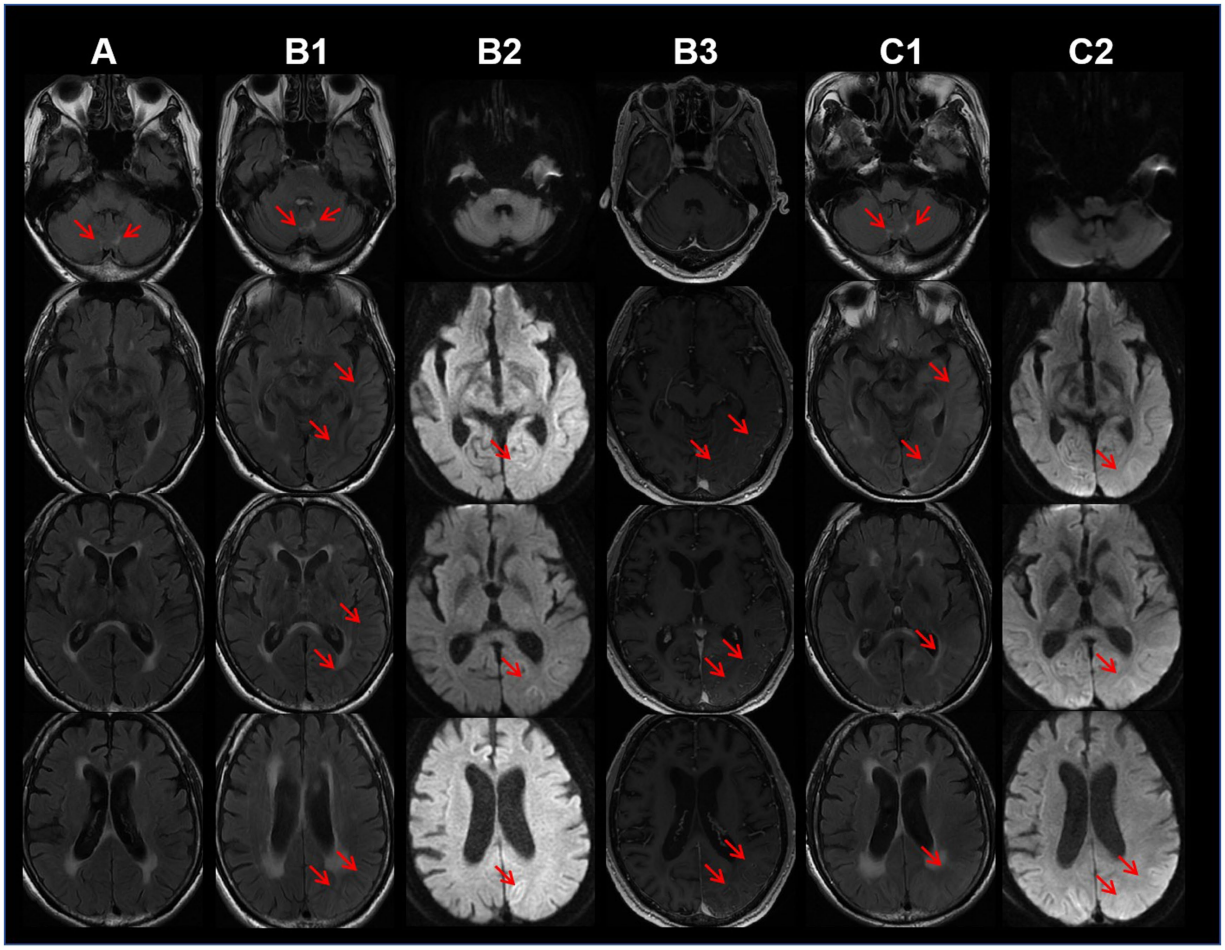
Column A: 22 months before dementia onset, the axial T2-weighted fluid attenuated inversion recovery (FLAIR) MRI sequence shows bilateral hyperintensities in the cerebellar paravermal region. Column B: Ten days after dementia onset, FLAIR sequence (B1) shows edema in the temporo-occipital lobes, diffusion-weighted imaging (DWI) (B2) reveals mildly restricted diffusion in the lesions, and contrast-enhanced MRI (B3) shows mild enhancement of the lesions. Column C: One month follow-up after discharge (52 days after symptom onset), FLAIR sequence (C1) demonstrates cortical edema and subcortical white matter hyperintensities, while DWI (C2) shows mildly restricted diffusion in the lesions.

The MRI showed swelling and contrast enhancement in the left temporal–parietal-occipital lobe, hippocampus and leptomeninges. Symmetrical white matter hyperintensities (WMHs) around the ventricles and in the paravermal region of cerebellum was found on T2WI and FLAIR imaging. DWI revealed slightly restricted diffusion in the cortical edema area. The lesions on the DWI did not exhibit the wedge-shaped restricted (Figure 1) diffusion affecting both the cortex and white matter typically observed in ischemic strokes. Moreover, these lesions also did not conform to the perfusion territory of a single major intracranial artery. Additionally, CT angiography of the head and neck showed no stenosis in the perfusion arteries of the lesions, and cardiac ultrasound did not identify any lesions that could cause embolism. These imaging results do not support the diagnosis of acute cerebrovascular disease. It is noteworthy that lesions in the paravermal region of cerebellum was already present 22 months before the onset of cognitive decline (Figure 1). The electroencephalogram (EEG) demonstrated severe 4–6 Hz theta waves and 2–3 Hz high-amplitude delta rhythms emanating from the left pontine, indicating abnormal brain activity.

Cerebrospinal fluid test revealed normal white cell count, slightly elevated protein (480 mg/L), negative results for bacterial and fungal cultures, acid-fast and ink staining, virus IgM antibodies, and Gene-Xpert test for tuberculosis. Blood and cerebrospinal fluid examinations showed negativity for autoimmune encephalitis and paraneoplastic syndrome antibodies.

**Figure 2:**
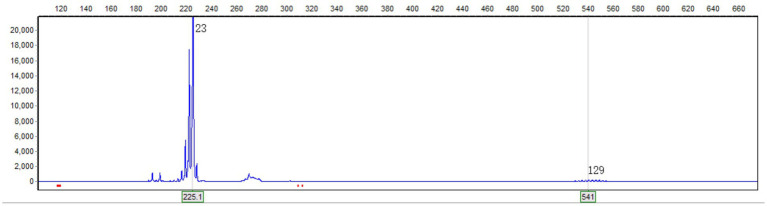
Genetic testing revealed a GGC repeat count of 129 in one of the alleles of the NOTCH2NLC gene. The horizontal axis represents the length of the nucleic acid strand, and the vertical axis represents the relative abundance of the nucleic acid.

CJD can damage the cortex without systemic inflammatory manifestations. However, the patient’s symptoms progressed extremely rapidly (within 1 day), and there were no muscle spasms. Contrast-enhanced MRI revealed lesions enhancement, and hyperintensities in the cerebellar paravermal region on FLAIR imaging existed 22 months prior to dementia onset. Additionally, there was an absence of the basal ganglia hyperintensities on DWI and the triphasic waves on the EEG. These pieces of evidence do not support the diagnosis of CJD. On the other hand, genetic disorders like MELAS syndrome may manifest as acute cortical edema. However, the peak age of onset for MELAS syndrome is before 20 years, only 1–6% patients over 40 years old, typically accompanied by exercise intolerance, sensorineural hearing loss, migraine, and intellectual disability (7). This patient is 60 years old, without maternal family history of myopathy or encephalopathy. He was a farmer and the primary breadwinner for the family before admission, and had no prior intellectual or physical impairments as confirmed by family members, aside from nonspecific headaches. Moreover, the hyperintensities on the FLAIR imaging in the cerebellar paravermal region are not characteristic of MELAS syndrome. These clinical features do not align with MELAS syndrome. Similar to MELAS syndrome, cortical edema can also occur in NIID. The NIID diagnosis was confirmed by genetic testing revealing an expansion of 129 repeats in the GGC segment of the NOTCH2NLC gene, while there have been no reported cases of confirmed CJD or MELAS syndrome patients with abnormalities in this gene (Figure 2). The method used for genetic testing was fluorescent PCR-capillary electrophoresis. However, the patient’s relatives refused further genetic testing for themselves.

After receiving 1 week of Huperzine A and mannitol therapy, the patient experienced a modest cognitive function recovery, including recognizing individuals and objects. During the weeks awaiting genetic testing results, we could not rule out the possibility of the autoimmune disease caused by newly identified antibodies. Concerned that delaying treatment might lead to irreversible brain damage, we recommended intravenous immunoglobulin (IVIG) therapy at the dose of 2 g per kilogram of body weight. With the consent of the patient and his family, the treatment was administered at other hospital. However, no substantial improvement was observed in cognitive status post-treatment. One month post-discharge, a follow-up MRI at our institution showed persistent cortical edema consistent with the previous results (Figure 1). Memory, orientation, and calculation abilities remained poor. During a telephone follow-up at 6 months post-discharge, the relative reported a significant improvement in cognitive function about 40 days after leaving our hospital. Spatial and personal orientation had recovered, enabling independent living and social engagement, with mild residual memory impairment and delayed responses. An 18-month telephone follow-up revealed the patient’s cognitive and physical functions had nearly returned to normal. The patient reported no further episodes of encephalopathy-like dementia since discharge. The disease progression of this patient, as indicated by follow-up, further ruled out the CJD diagnosis.

## Discussion

We report a patient with NIID who presented with sudden-onset dementia induced by acute cerebral cortical edema. The patient had already exhibited symptoms and typical imaging features of NIID 22 months before the onset of acute dementia. In this case, the patient declined the skin biopsy, the diagnosis was confirmed through genetic testing. Recent studies have shown that eosinophilic intranuclear inclusions observed in skin biopsies can also be found in other nucleotide repeat expansion diseases, such as oculopharyngeal distal myopathy (OPDM) (8) and fragile X-associated tremor/ataxia syndrome (FXTAS) (9), which reduces the specificity of skin biopsy in diagnosing NIID. Recent NIID study emphasize the combination of genetic abnormalities with clinical and imaging features as diagnostic criteria (10). Moreover, there were no individuals with neurological disorders in the family history, and the family members declined genetic testing. Therefore, a preliminary assessment suggests it’s the sporadic form of NIID. Inclusions form extensively in the central and peripheral nervous systems, skin, and visceral organs in NIID (11), contributing to a heterogeneous clinical presentation. Acute dementia episode and encephalitis-like imaging of this case account for approximately one-fifth of all NIID patients (5). The normal physiological range of GGC repeats in NOTCH2NLC gene is 7–40 (12). The diagnostic threshold for NIID is 60 (10, 12), with over 200 repeats being associated with the myopathic phenotype of NIID, 100–200 repeats linked to the dementia phenotype, and fewer than 100 repeats potentially resulting in the Parkinsonian phenotype (13), which aligns with the genetic characteristics observed in this case.

In recent years, the understanding of imaging features of NIID has continued to evolve. The hyperintensities at the corticomedullary junction on DWI, regarded as a hallmark of NIID, may not manifest until several years after the onset of major symptoms or can even completely disappear during the progression (14). Additionally, this imaging feature may also be observed in other diseases, such as FXTAS (15). Tian et al. (5) summarized the imaging features of 212 NIID patients as follows: 1. hyperintensities at the corticomedullary junction on DWI (89.6%), also known as the subcortical band sign; 2. hyperintensities in the corpus callosum on DWI (46.7%); 3. bilaterally symmetrical and extensive hyperintensities in the white matter on T2-weighted and FLAIR imaging (83.1%); 4. symmetric white matter lesions (WMLs) in the cerebellar paravermal region (88.7%) and middle cerebellar peduncle (35.2%); and 5. rare instances of localized cortical edema and contrast enhancement on MRI, this feature tends to manifest prior to the hyperintensities observed at the corticomedullary junction on DWI. The cortical edema coupled with the absence of lesions at the corticomedullary junction of this case, represents an unusual manifestation compared to previous reports, which may be associated with localized cerebral blood flow augmentation and increased blood–brain barrier permeability (16). Importantly, this case emphasizes the necessity of considering NIID in patients with acute dementia symptoms and imaging features resembling MELAS syndrome, even in the absence of a familial history of similar episodes.

It is noteworthy that 22 months before the dementia onset, the patient sought outpatient consultation for chronic headaches. A bilateral symmetric WMHs in the cerebellum paravermal region was already evident on the initial MRI (Figure 1), which is one of the imaging marks of NIID (17). Unfortunately, the primary physician did not pursue further investigations, attributing the symptom to “the tension-type headache.” Previous case reports indicate that approximately 10.9% of NIID patients have chronic headaches (5). The mechanism may be related to vascular dysfunction caused by eosinophilic inclusions (18). Some researchers propose that migraine and encephalopathy-like episodes could represent manifestations of different stages of NIID (19). Considering that the cerebellar paravermal region is an uncommon location for vascular-origin WMLs, while it may be a long-term clue of NIID during non-encephalopathic phases, this case underscores the importance for clinicians to contemplate the NIID in patients exhibiting chronic headaches plus symmetric cerebellar WMHs on MRI.

There is currently no established evidence for effective treatment strategies for NIID, and the therapy primarily focuses on symptomatic relief. The effectiveness of corticosteroids is under contention. Corticosteroid pulse therapy helps alleviate brain edema and improve consciousness but does not repair cognitive impairment. The long-term effects of steroids remain unclear (1). Many NIID patients with encephalitis-like episodes have been reported to spontaneously improve within days to weeks with or without undergoing Immunomodulatory therapy (20, 21). In this case, despite receiving IVIG treatment at a dose of 2 g/kg body weight, cognitive impairment and imaging lesions did not show improvement upon 1 month follow-up. However, spontaneous symptom improvement occurred approximately 1 month after IVIG therapy, prompting a cautious stance on the efficacy of IVIG. Further study is needed to explore the risk factors and mechanisms underlying the heterogeneous clinical phenotype and prognosis of this disease.

## Data Availability

The original contributions presented in the study are included in the article/Supplementary material, further inquiries can be directed to the corresponding author.
