# Supplementary material for: Neuronal Intranuclear Inclusion Disease Presenting with Acute-Onset Dementia and Cortical Edema: A Case Report
Source: Front Neurol. 2024 Oct 28;15:1464991. doi: 10.3389/fneur.2024.1464991 (PMC11551014; doi:10.3389/fneur.2024.1464991)
Supplement: Supplementary file 1 [file Data_Sheet_1.pdf]

CARE Checklist of information to include when writing a case report

CARE case report guidelines

(cc) BY-NC-ND

Topic	Item	Checklist item description	Reported on Line
Title	1	The diagnosis or intervention of primary focus followed by the words "case report"	page 1, title
Key Words	2	2 to 5 key words that identify diagnoses or interventions in this case report, including "case report"	page 1, keywords
Abstract (no references)	3a	Introduction: What is unique about this case and what does it add to the scientific literature?	page1-2, introduction
	3b	Main symptoms and/or important clinical findings	page 2-4, case presentation
	3c	The main diagnoses, therapeutic interventions, and outcomes	page 3-4, case presentation
	3d	Conclusion—What is the main "take-away" lesson(s) from this case?	page 4, discussion
Introduction	4	One or two paragraphs summarizing why this case is unique (**may include references**)	page 4, discussion
Patient Information	5a	De-identified patient specific information.	page 2, case presentation
	5b	Primary concerns and symptoms of the patient.	page 2, case presentation
	5c	Medical, family, and psycho-social history including relevant genetic information	page 2, case presentation
	5d	Relevant past interventions withoutcomes	page 2, case presentation
Clinical Findings	6	Describe significant physical examination (PE) and important clinical findings.	page 2, case presentation
Timeline	7	Historical and current information from this episode of care organized as a timeline	page 2-4, case presentation
Diagnostic Assessment	8a	Diagnostic testing (such as PE, laboratory testing, imaging, surveys).	page 2-3, case presentation
	8b	Diagnostic challenges (such as access to testing, financial, or cultural)	page 2, case presentation
	8c	Diagnosis (including other diagnoses considered)	page 2-3, case presentation
	8d	Prognosis (such as staging in oncology) where applicable	page 4, case presentation
Therapeutic Intervention	9a	Types of therapeutic intervention (such as pharmacologic, surgical, preventive, self-care)	page 4, case presentation
	9b	Administration of therapeutic intervention (such as dosage, strength, duration)	page 4, case presentation
	9c	Changes in therapeutic intervention (with rationale)	page 4, case presentation
Follow-up and Outcomes	10a	Clinician and patient-assessed outcomes (if available)	page 2, case presentation
	10b	Important follow-up diagnostic and other test results	page 2-3, case presentation
	10c	Intervention adherence and tolerability (How was this assessed?)	page 4, case presentation
	10d	Adverse and unanticipated events	page 2, case presentation
Discussion	11a	A scientific discussion of the strengths AND limitations associated with this case report	page 4, discussion
	11b	Discussion of the relevant medical literature **with references**.	page 4, discussion
	11c	The scientific rationale for any conclusions (including assessment of possible causes)	page 4, discussion
	11d	The primary "take-away" lessons of this case report (without references) in a one paragraph conclusion	page 4, discussion
Patient Perspective	12	The patient should share their perspective in one to two paragraphs on the treatment(s) they received	The patient lacks this ability
Informed Consent	13	Did the patient give informed consent? Please provide if requested	Yes ☑ No ☐
